# Peak event analysis: a novel empirical method for the evaluation of elevated particulate events

**DOI:** 10.1186/1476-069X-12-92

**Published:** 2013-11-01

**Authors:** Aaron Orkin, Pamela Leece, Thomas Piggott, Paul Burt, Ray Copes

**Affiliations:** 1 Dalla Lana School of Public Health, University of Toronto, 155 College Street, Toronto, ON M5S 3 M2, Canada; 2 Michael G. DeGroote School of Medicine, McMaster University, Hamilton, Canada; 3 Ministry of the Environment, Kingston, Canada; 4 Public Health Ontario, Environmental Health, Toronto, Canada

**Keywords:** Air quality, Suspended particulate matter, Public health, Environmental health, Peak event analysis, Dust

## Abstract

**Background:**

We report on a novel approach to the analysis of suspended particulate data in a rural setting in southern Ontario. Analyses of suspended particulate matter and associated air quality standards have conventionally focussed on 24-hour mean levels of total suspended particulates (TSP) and particulate matter <10 microns, <2.5 microns and <1 micron in diameter (PM_10_, PM_2.5_, PM_1_, respectively). Less emphasis has been placed on brief peaks in suspended particulate levels, which may pose a substantial nuisance, irritant, or health hazard. These events may also represent a common cause of public complaint and concern regarding air quality.

**Methods:**

Measurements of TSP, PM_10_, PM_2.5_, and PM_1_ levels were taken using an automated device following local complaints of dusty conditions in rural south-central Ontario, Canada. The data consisted of 126,051 by-minute TSP, PM_10_, PM_2.5_, and PM_1_ measurements between May and August 2012. Two analyses were performed and compared. First, conventional descriptive statistics were computed by month for TSP, PM_10_, PM_2.5_, and PM_1_, including mean values and percentiles (70th, 90th, and 95th). Second, a novel graphical analysis method, using density curves and line plots, was conducted to examine peak events occurring at or above the 99th percentile of per-minute TSP readings. We refer to this method as “peak event analysis”. Findings of the novel method were compared with findings from the conventional approach.

**Results:**

Conventional analyses revealed that mean levels of all categories of suspended particulates and suspended particulate diameter ratios conformed to existing air quality standards. Our novel methodology revealed extreme outlier events above the 99th percentile of readings, with peak PM_10_ and TSP levels over 20 and 100 times higher than the respective mean values. Peak event analysis revealed and described rare and extreme peak dust events that would not have been detected using conventional descriptive statistics.

**Conclusions:**

Peak event analysis underscored extreme particulate events that may contribute to local complaints regarding intermittently dusty conditions. These outlier events may not appear through conventional analytical approaches. In comparison with conventional descriptive approaches, peak event analysis provided a more analytical and data-driven means to identify suspended particulate events with meaningful and perceptible effects on local residents.

## Background

Elevated suspended air particulate levels are associated with increased cardiovascular and respiratory mortality [1]. These associations have been widely recognized in settings where populations experience ongoing exposure to suspended particulates arising from human activities and combustion [1]. More recently, elevated mortality has been associated with naturally-occurring suspended particulates and relatively brief exposures, such as Saharan dust blown to the Iberian Peninsula [2]. In occupational settings, brief and even singular exposures to high levels of irritant dust, vapour, fume or smoke has, in patients with no prior history of respiratory disease, been described as the causative agent in reactive airways dysfunction syndrome and irritant-induced asthma, resulting in symptoms of airway inflammation and bronchial reactivity without a latency period [3,4].

Canadian air quality standards have conventionally focussed on mean suspended particulate values measured over multiple years. The Canadian Ambient Air Quality Objective is 120 μg/m^3^, measured as a 3-year mean of 24-hour mean total suspended particulate (TSP) levels. Even subtle changes in a 3-year mean TSP value will likely have meaningful aggregate health effects, but are unlikely to result in immediately perceptible changes to residents or complaints regarding air quality [5].

In contrast, relatively brief peak events in suspended particulate matter may be readily perceptible or represent a nuisance and cause for complaint for people residing in a given area, and may result in discomfort and mucous membrane and airway irritation. Canadian standards identify 1-hour mean limits for sulphur dioxide, ozone, nitrogen dioxide, and hydrogen sulphide, but do not place limits on relatively brief peak allowances for PM_2.5_ or PM_10_ by defining limits at the 98th percentile or through 24-hour and annual means [6]. Air quality standards in other jurisdictions do identify peak limits. United States standards, for example, indicate that 24-hour PM_10_ readings are not to exceed 150 μg/m^3^ more than once per year over 3 years [7]. However, infrequent peak events lasting only a few hours may not be identifiable through the analysis of 24-hour means.

This paper concerns the analysis of air quality data collected in a rural Ontario community, following local complaints concerning episodic dusty conditions to determine if the available data confirmed the occurrence of episodic peaks in particulate levels. A 31 August 2012 public statement from the County Health Unit identified the dust source as a tailings pile from an upwind nepheline syenite mine [8]. We describe a novel approach to suspended particulate data analysis, designed to identify and explore peak dust events irrespective of whether those events produce deviations from regulatory air quality standards.

## Methods

### Suspended particulate data

In response to local complaints of dusty conditions, from 24 May 2012 to 2 October 2012, the Ontario Ministry of the Environment conducted automated suspended particulate and basic meteorological measurements at the test site, located 2.5 km and 5.5 km from two nepheline syenite mine processing locations. The survey design was to verify the complaints and try to determine the source. A real-time aerosol particulate analyzer, (Grimm Technologies Inc., Germany, Model 107) was used for this survey. Instrument intake consisted of a stainless steel PM_10_ head and inlet located approximately 6 feet off the ground. The instrument had been calibrated before then after the survey. The survey instruments produced minute readings of particulate matter less than 10 microns, 2.5 microns and 1 micron in diameters (PM_10_, PM_2.5_, and PM_1_ respectively) and computed readings of total suspended particulates (TSP), as well as humidity, barometric pressure, wind speed and wind direction.

The data set included 10 548 observations for May 2012, 26 705 for June 2012, 13 771 observations for July 2012, 29 595 observations for August 2012, 43 187 observations for September 2012 and 2245 observations for October 2012. Differences in the number of observations per month were due to variations in the amount of time when the monitoring equipment was deployed, and due to events such as power outages at the testing site.

### Analyses performed

We analysed these data from a public health perspective in order to determine if local air quality complaints could be traced to elevated particulate levels. Our analysis was conducted using two approaches. The first analysis followed conventional approaches drawn from Brook and colleagues’ large-scale study of Canadian atmospheric particulate matter [9]. For each of TSP, PM_10_, PM_2.5_, and PM_1_, the data were described by month according to the mean, maximum and minimum values, the standard deviation, and the 10th, 50th, 70th, 90th and 95th percentiles percentile readings for each suspended particulate measurement. These values were then compared with Canadian standards, assuming, according to the Central Limit Theorem, that the mean of the minute values would approximate the mean of 24-hour values. These results were also compared with combined summary statistics from 14 urban Canadian sites as reported by Brook and colleagues.

Our second analysis was designed to highlight peak events. A tenfold rise in suspended particulate levels lasting 2 hours per week (or 8 hours per month) may affect only the 99th percentile of measured suspended particulate values. Depending on other concurrent changes and sources of environmental particulates, these brief peak events may have minimal effect on calculated monthly means. Peak event analysis consisted of three analyses of particulate measurements above the 99th percentile. First, the presence of peak readings was confirmed by plotting smoothed density curves of the top 1% of values for each of TSP, PM_10_, PM_2.5_, and PM_1_. Second, summary quantile statistics were computed for each of TSP, PM_10_, PM_2.5_, and PM_1_ values by month: 99th percentile, and each permillile (‰ile) between 990 and 999. These permilliles permit assessment of peaks. Third, line graphs of per-minute TSP and PM_10_ values were generated for each month, to provide a visual analysis of peak events.

Our analysis was not designed to associate elevated particulate events with a given source. Analysis of meteorological data was not performed.

All statistical computations and visualizations were generated using the R Statistical Package v.2.15.1 and Microsoft Excel 2010.

## Results

### Conventional analysis

Table 1 provides a summary of descriptive statistics arising from the first conventional analysis. Median statistics show minimal variability by month. Median by-minute TSP ranges from 10.30 μg/m^3^ in October to 26.65 μg/m^3^ in May. Assessment of TSP values at the 95th percentile shows more variation, ranging from 20.20 μg/m^3^ in October to 144.47 μg/m^3^ in May. Similarly, PM_10_ values show minimal variability at the median (median of 8.80 μg/m^3^ in October to 16.20 μg/m^3^ in May, 95th percentile values of 13.00 μg/m^3^ in October to 54.30 μg/m^3^ in May). PM_2.5_ values show less variability at the median and 95th percentile (medians of 6.40 μg/m^3^ in October to 10.10 μg/m^3^ in July, 95th percentile values of 8.00 μg/m^3^ in October to 23.90 μg/m^3^ in May). PM_1_ values show still less variability at the median and 95th percentile (medians of 3.20 μg/m^3^ in October to 6.8 μg/m^3^ in July, 95th percentile values of 4.30 μg/m^3^ in October to 19.50 μg/m^3^ in May).

**Table 1:** Conventional by-minutes summary statistics PM1, PM2.5, PM10 and TSP, May-October 2012

	**Percentiles**					**Statistics**			
	**10th**	**50th**	**70th**	**90th**	**95th**	**Mean**	**Min**	**Max**	**SD**
PM_1_ μg/m^3^									
May	2.00	5.00	10.20	17.00	19.50	7.40	0.80	53.10	5.83
Jun	2.70	5.10	7.40	12.10	13.50	6.21	0.80	63.70	3.65
July	3.40	6.80	8.30	10.30	11.10	6.74	1.90	32.50	2.64
Aug	2.60	5.70	7.30	10.60	13.10	6.30	0.60	67.00	3.68
Sept	1.50	3.50	4.80	7.20	9.30	4.02	0.20	50.10	2.49
Oct	2.10	3.20	3.50	3.90	4.30	3.11	1.80	6.00	0.69
PM_2.5_ μg/m^3^									
May	4.60	8.50	13.80	21.10	23.90	10.92	2.70	85.00	6.84
Jun	5.50	8.30	10.90	15.80	17.20	9.50	2.90	68.00	3.99
July	6.20	10.10	11.70	13.80	14.60	9.93	4.30	36.90	2.90
Aug	5.50	9.00	10.60	14.40	17.10	9.64	2.30	136.90	4.82
Sept	4.00	6.70	8.10	10.80	12.90	7.12	0.90	88.80	2.94
Oct	4.90	6.40	6.70	7.40	8.00	6.29	4.30	10.20	0.99
PM_10_ μg/m^3^									
May	5.70	16.20	24.70	42.20	54.30	23.18	2.90	953.50	37.38
Jun	6.60	11.90	16.30	22.60	25.70	13.89	3.30	182.30	8.65
July	7.00	12.60	15.20	18.90	21.10	12.90	4.40	51.40	4.90
Aug	6.70	11.30	14.00	20.40	24.80	13.90	2.30	800.40	11.32
Sept	4.60	9.00	12.00	17.00	20.10	10.30	0.90	931.60	9.52
Oct	6.60	8.80	9.80	11.50	13.00	9.06	4.70	32.00	2.22
TSP μg/m^3^									
May	6.50	26.65	46.90	97.50	144.47	47.73	3.10	1,802.00	89.25
Jun	7.80	16.20	25.10	38.80	47.40	23.13	3.50	5,958.00	63.33
July	8.50	17.70	21.90	28.30	32.30	18.36	5.10	115.10	8.87
Aug	7.90	15.40	19.80	32.60	46.10	45.15	2.50	11,210.00	373.11
Sept	5.00	10.90	15.50	23.80	29.00	13.66	1.00	1782.00	19.42
Oct	7.60	10.30	11.60	15.20	20.20	11.31	5.30	61.30	4.93

TSP: Total Suspended Particulates, PM: particulate matter, SD: Standard Deviation.

Overall, using the 10th, 50th, 70th, 90th and 95th percentiles for analysis, May appears to be by far the dustiest month, while readings in October show substantially less suspended particulate. Variation in the months of June to September appears to be less substantial.

These statistics provide some suggestion of extreme peak levels, especially in PM_10_ and TSP values. The maximum value for TSP in August is 11,210.00 μg/m^3^*,* with a standard deviation more than fourfold higher than any other month. In May, maximum PM_10_ levels are 953.50 μg/m^3^ with a standard deviation more than threefold greater than other months. This conventional analysis does not provide further insights or description of these peak events, though it suggests data heavily skewed toward the lower end of a wide distribution.

### Peak event analysis

**Figure 1:**
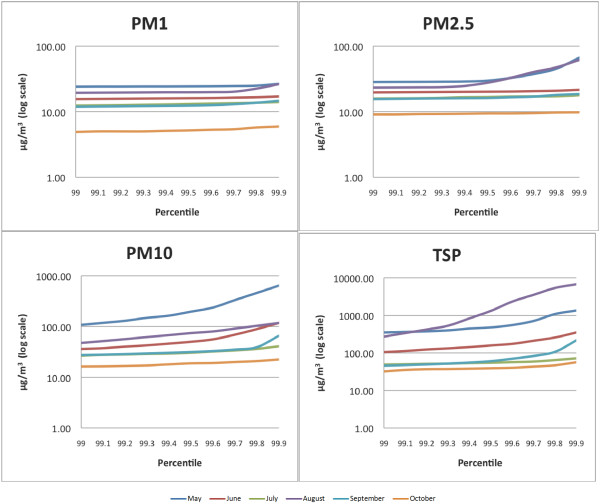
Graphs of 990th to 999th permillile of PM1, PM2.5, PM10 and TSP readings.

Figure 1 provides by-month curves of TSP, PM_10_, PM_2.5_, and PM_1_ readings at and above the 99th percentile of readings (or the 990th to 999th permillile of readings). Note that the y-axis is displayed on a logarithmic scale. These graphs portray trends in rare peak measurements in suspended particulate levels. Between the 990th and 999th permillile, substantial variability is observed that cannot be captured even by analysing the 95th or 99th percentile of readings. This provides evidence of substantial, but brief, peaks in the observed levels of suspended particulates, especially TSP and PM_10_.

These effects are particularly pronounced in May and August. In May, where the 99th percentile corresponds with 105 observations, peak levels of TSP occurring at the 999th permillile are more than 3.8 times higher than those levels observed at the 990th permillile, while peak levels of PM_10_ at the 999th permillile are 6.0 times higher than those levels observed at the 990th permillile. In August, where the 99th percentile corresponds with 295 observations, peak levels of TSP occurring at the 999th permillile are more than 24.9 times higher than those levels observed at the 990th permillile, while peak levels of PM_10_ at the 999th permillile are more than 2.5 times higher than those levels observed at the 990th permillile. In both May and August, peak events in PM_2.5_ are also observed above the 994th permilile. None of these effects are seen through the conventional analysis. In comparison with May and August, there is less evidence of extreme variation in the top percentile of the suspended particulate data in June, July, September and October. Given that the 90th and 95th percentile values were also not elevated in comparison with mean and median values in these months, there were therefore fewer brief peak events in June, July, September and October.

**Figure 2:**
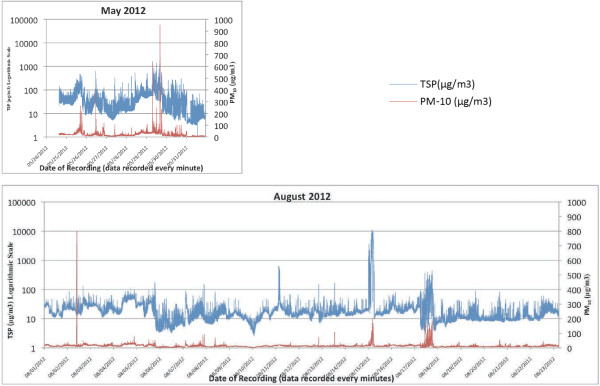
**Graphical analysis of TSP and PM**_**10 **_**data over time.**

Figure 2 provides exemplars of the line graphs generated to further explore the peak events observed in May and August. In May, peak events in TSP are accompanied closely by elevations in PM_10_. In August, however, some substantial elevations in TSP that contribute to the highest percent of readings are not accompanied by proportionate PM_10_ elevations (for example, the TSP spike on 8 August 2012 and on the first half of 15 August 2012).

## Discussion

These two analyses reveal different features in the same data set and provide an opportunity to compare the information derived from conventional evaluations of suspended particulate with the information arising from our novel peak event analysis.

Our initial approach to suspended particulate data analysis, identified here as the conventional approach, succeeded in providing adequate information to confirm that the air quality at the test site likely conforms with Canadian air quality standards. (The data could not confirm this conclusively because a 3-year mean of 24-hour values cannot be computed.) Furthermore, our conventional approach permits comparison to other dominant literature in this field. Brook and colleagues' study of suspended particulates in 14 urban Canadian centres provides an appropriate national comparator. In their study, mean TSP and PM_10_ values were 46.0 and 24.0, respectively. In the same study, TSP and PM_10_ values at the 95th percentile were found to be 123.0 and 58.0, respectively. In all months at the test site, mean monthly values for TSP, PM_10_ and PM_2.5_ were below mean 24-hour values reported by Brook and colleagues. Table 2 lists these comparators.

**Table 2:** **Summary 24-hour statistics from 14 urban sites, adapted from Brook et al., 1997**[9]

	**Percentiles**					**Statistics**		
	**10th**	**50th**	**70th**	**90th**	**95th**	**Mean**	**Max**	**SD**
TSP	22.0	46.0	62.0	98.0	123.0	55.2	572.0	37.8
PM_10_	11.3	24.0	32.0	47.4	58.0	27.6	175.0	16.3
PM_2.5_	5.0	11.0	16.0	26.1	32.2	13.9	89.0	9.5

TSP: Total Suspended Particulates, PM: particulate matter, SD: Standard Deviation.

However, our conventional analysis also reveals the limitations of this approach. Local concerns regarding air quality in the community – and indeed the impetus for initiating air quality monitoring at this site – pertained to peak events, not changes in baseline suspended particulate levels. Immediate community concerns related to eye irritation, nose and upper airway irritation, and difficulty breathing [5], not long-term impacts on cardiorespiratory health. Peak suspended particulate events may not have any identifiable effect on mean annual suspended particulate measurements or result in a departure from standards based on 3-year means. Our peak event analysis centered on a statistical interrogation of suspended particulate readings at or above the 99th percentile of readings. Above the 99th percentile there is evidence that substantial TSP peaks occurred with particular severity in May and August. With the exception of August, all months after May showed substantially fewer peak episodes. In some cases, and with per-minute data like that used in this study, peak episodes may be observed as readings above the 995th permillile, and may not affect that 90th, 95th or 99th percentile values.

Some of the very high TSP levels observed in this study may be due the measurement instrument errors. Particles above 10 microns can interact with the instrument’s laser and sensor to over-report TSP concentrations under certain conditions. This effect is most likely to occur under high relative humidity conditions because the instrument has a limited capacity to dry the incoming air stream [10]. Under long periods of high humidity conditions this capacity can be exceeded. High humidity conditions can cause unusual TSP readings under certain conditions. Even the highest TSP measurements reported fall within the instrument’s measurement range.

The Ontario occupational exposure limit for the respirable fraction of nepheline syenite—the primary mining product at the upwind site and primary contributor to the particulate measurements herein—is 10 mg/m^3^, or 10,000 μg/m^3^[11]. American occupational permissible exposure limits are half this figure (5 mg/m^3^) [12]. This exposure limit is intended for an occupational setting, and not a general outdoor setting. Brief episodes of TSP readings well above 1000 μg/m^3^ will likely pose a substantial and perceptible nuisance and may have aggregate health effects. Whether mean 24-hour TSP, PM_10_ or PM_2.5_ readings are below existing standards is not relevant to this finding. Conventional analyses of suspended particulate data may not attend to peak events.

## Conclusions

This analysis shows minimal variation in mean TSP, PM_10_, PM_2.5_ and PM_1_ levels between May and October 2012. Throughout this period, mean TSP readings matched closely with Canadian air quality standards. However, these standards do not attend to measurements of extreme peaks in dust levels or their potential health effects. Such peaks are observed in the data provided, particularly in August and May 2012. Identifying these peak events require a different approach to suspended particulate data analysis. The method presented here identifies peak events occurring at or above the 99th percentile of by-minute suspended particulate data. This approach provides a data-driven way to identify and explore peak events without relying on the use of an arbitrary cut-point for peaks. Peak event analysis may complement conventional approaches to suspended particulate analysis, and may provide opportunities to identify airborne hazards to human health that would escape more conventional analyses.

## Abbreviations

TSP: Total suspended particulates; PM10: PM_2.5_, PM_1_, Particulate matter less than 10, 2.5 and 1 microns in diameter, Respectively.

## Competing interests

The authors declare that they have no competing interests.

## Authors’ contributions

AO conceived of and designed and carried out the statistical analysis for the study and drafted the manuscript. PL participated in the design of the study and helped to draft the manuscript. TP contributed to preparation of the data graphical representation and helped to draft the manuscript. PB contributed to air quality data collection and technical interpretation of the air quality instrument’s measurements. RC participated in the study design and helped to draft the manuscript. All authors read and approved the final manuscript.

## References

[B1] Committee on the Medical Effects of Air Pollutants (Chair: JG Ayres) Long-term exposure to air pollution: Effect on mortality 2009 London

[B2] Jimenez E Linares C Martinez D Diaz J Role of Saharan dust in the relationship between particulate matter and short-term daily mortality among the elderly in Madrid (Spain) Sci Total Env 2010 408 5729 5736 10.1016/j.scitotenv.2010.08.049 20855107

[B3] Brooks SM Weiss MA Bernstein IL Reactive airways dysfunction syndrome Chest 1985 88 376 384 10.1378/chest.88.3.376 4028848

[B4] Labrecque M Irritant-induced asthma Curr Opin Allergy Clin Immunol 2012 12 140 144 10.1097/ACI.0b013e32835143b8 22327170

[B5] Canadian Counsel of Ministers of the Environment Canada-Wide Standards for Particulate Matter (PM) and Ozone 2000 http://www.ccme.ca/assets/pdf/pmozone_standard_e.pdf

[B6] Environment Canada National Ambient Air Quality Objectives 2010 http://www.hc-sc.gc.ca/ewh-semt/pubs/air/naaqo-onqaa/index-eng.php

[B7] International Air Quality Advisory Board Progress Report 25 for the International Joint Commission (Chapter 5) 2000 http://www.ijc.org/php/publications/html/progress25/chap5.html

[B8] Pellizzari R Message from the Medical Officer of Health Peterborough County-City Health Unit Friday, August 31, 2012 2012

[B9] Brook JF Dann TF Burnett RT The relationship Among TSP, PM10, PM2.5, and Inorganic Constituents of Atmospheric Particulate Matter at Multiple Canadian Locations J Air Waste Manage Assoc 1997 47 2 19

[B10] United States Environmental Protection Agency Clean Air Scientific Advisory Committee Ambient Air Monitoring and Methods Subcommittee Review of the White Paper on Particulate Matter (PM) Light Extinction Measurements 2010 http://yosemite.epa.gov/sab/sabproduct.nsf/92C9F5AA09A76A93852577150004A782/$File/EPA-CASAC-10-010-unsigned.pdf

[B11] Ontario Ministry of Labour Occupational Exposure Limits for Ontario Workplaces 2013 http://www.labour.gov.on.ca/english/hs/pubs/oel_table.php

[B12] Centers for Disease Control National Institute for Occupational Health and Safety Particulates Not Otherwise Specified 2012 http://www.cdc.gov/niosh/npg/npgd0480.html

